# Case Report: Vitamin C combined with multiple micronutrient deficiencies is associated with pulmonary arterial hypertension in children with autistic spectrum disorder

**DOI:** 10.3389/fnut.2022.928026

**Published:** 2022-10-20

**Authors:** Wirada Sakamornchai, Oraporn Dumrongwongsiri, Sirinapa Siwarom

**Affiliations:** Division of Nutrition, Department of Pediatrics, Faculty of Medicine Ramathibodi Hospital, Mahidol University, Bangkok, Thailand

**Keywords:** vitamin C deficiency, autistic spectrum disorder, pulmonary arterial hypertension, micronutrient deficiencies, inappropriate feeding problems

## Abstract

Children with developmental and behavioral problems including autistic spectrum disorders (ASDs) may have inappropriate feeding behaviors, which leads to an increased risk of multiple nutrient deficiencies. Vitamin C deficiency is one of the common nutrient deficiencies reported in children with inappropriate feeding. This case report illustrates two cases of ASD children with a clinical presentation of pulmonary arterial hypertension, a rare presentation of vitamin C deficiency. Vitamin C supplementation, pulmonary vasodilator, and supportive treatment were provided. Patients could recover from the illness and could be discharged from the hospital in a short time. In addition to vitamin C, the patients also had multiple micronutrient deficiencies. Nutrition counseling was given and micronutrient supplement was continued until follow-up. Regular nutrition assessment and counseling among children with ASD are needed to prevent nutrient deficiencies which may lead to life-threatening complications.

## Introduction

Inappropriate feeding behaviors are common in children who have developmental and behavioral issues and they increase the risk of nutrient deficiencies. Vitamin C deficiency was frequently reported in children with highly selective food intake. The common clinical presentations of vitamin C deficiency are joint pain, refuse to walk, and bleeding tendency (1). In this case report, we summarized the clinical presentations of two vitamin C-deficient autistic spectrum disorder (ASD) children who presented with pulmonary arterial hypertension (PAH), which is a rare presentation of vitamin C deficiency.

## Case presentation 1

A 6-year-old boy with a history of ASD, presented with progressive dyspnea for 1 day. He had progressive bilateral knee swelling and refused to walk for 2 months. His symptoms were not improved by analgesics. Dietary history showed that his meals consisted of rice porridge and boiled egg. Some biscuits were provided as a snack. He never drank milk for 2 years and refused red meat, fruits, and vegetables.

At the emergency department, his respiratory rate was 48 breaths per minute, heart rate was 130 beats per minute, blood pressure was 107/78 mmHg, and he had low oxygen saturation at room air (SpO_2_ 90%) that could be corrected with oxygen *via* face mask 10 LPM. His weight, length, and BMI were 20 Kg, 112 cm and 15.94 Kg/m^2^, respectively (weight-for-age Z-score; WAZ –0.23, height-for-age Z-score; HAZ –0.86, BMI-for-age Z-score 0.46 according to the WHO growth standard). Physical examination showed that he was pale and had respiratory distress. Neither scorbutic nor rachitic rosary was seen on chest wall. Left leg was swelling and limited movement due to pain. Corkscrew hair was presented over the body. Other physical examinations were unremarkable. Initial laboratory investigations are shown in Table 1. Echocardiogram showed tricuspid regurgitation (TR) with tricuspid regurgitation pressure gradient (TRPG) of 80 mmHg and D-shape left ventricle (LV). He was diagnosed with severe PAH. No evidence of other causes of PAH such as congenital heart disease, pulmonary embolism, and malignancy was found. He was stabilized in the pediatric intensive care unit. Respiratory support, inotropic drugs, and pulmonary vasodilator were given. Daily 300 mg of vitamin C was given *via* oral route since the first day of admission. He gradually recovered from respiratory distress and could be discharged in 13 days. Due to improper dietary intake, nutrition counseling was given. The patient was encouraged to take the medical formula as an oral nutritional supplement (ONS) to get optimal micronutrient intake. Micronutrient supplements (multivitamins, vitamin C, vitamin D, iron, and folate) and ONS were continued after discharge. One-month follow-up visit after discharge showed that he was able to mobilize both lower extremities without pain and had no dyspnea while doing the activity. He could have more diet variation but still refused fruits and vegetables. ONS was stopped because of patient refusal and more variation in dietary intake. Micronutrient supplement was continued. Echocardiogram at 2 months after discharge showed no evidence of PAH and the pulmonary vasodilator could be weaned off.

**TABLE 1:** Biochemical parameters at diagnosis and follow-up visit.

Parameter	Normal value	Case 1		Case 2	
		At diagnosis	Follow up^1^	At diagnosis	Follow up^1^
Hemoglobin (g/dL)	12.5 (11.5) ^†^ 13.5 (11.5) ^‡^	7.0	9.4	9.9	11.7
Mean corpuscular value (fL)	81 (75) ^†^ 86 (77) ^‡^	60	77.8	54.2	65.7
Serum iron (mcg/dL)	50–120	14	101	20	63
Total iron-binding capacity (mcg/dl)	100–400	313	322	500	421
Transferrin saturation (%)	≥16%	4.4	31.3	4	15
Ferritin (ng/ml)	7–140	41.5	88.9	7.5	25.3
25-OHD (ng/ml)	20–100	16.2	64.9	6.02	NA
Vitamin C (mg/L)	0.4–2	Undetectable	10.9	Undetectable	NA
Thiaminepyrophosphate (mcg/L)	28–85	NA	NA	49.2	NA
Serum folate (ng/ml)	4–20	4.6	NA	3.2	1132.9
Copper (mcg/dL)	110–145	NA	NA	131	NA
Zinc (mcg/dL)	75–120	NA	NA	65	122

^†^Data are mean (-2 SD) for age 2 to <6 year; ^‡^Data are mean (-2 SD) for age 6 to <12 year; ^1^Follow-up visit at 1 month after discharge in Case 1 and 3 weeks after discharge in Case 2 NA; not available.

## Case presentation 2

A 5-year-old boy with a history of ASD, allergic rhinitis, and snoring, presented with progressive dyspnea and refuse to walk for 2 weeks. He also had a history of bleeding per gum 3 weeks ago. Dietary history showed that he had only rice porridge without any meat for 1 year, and he just started having some amount of boiled egg for 1 week. He usually drank 12 cartons (6 oz. per carton) of plain UHT milk per day *via* bottle feeding. He always refused fruits and vegetables and did not receive any vitamin or mineral supplementation.

At the outpatient unit, his respiratory rate was 50 breaths per minute, he had no fever, his heart rate was 136 beats per minute, and his blood pressure was 110/60 mmHg. His weight and height were 30.2 Kg and 115 cm, respectively. (WAZ 3.01, HAZ 0.2, BMI-for-age Z-score 4.14). He had mildly pale conjunctivae, dental caries, and bleeding per gum. No scorbutic rosary and corkscrew hair was seen. There were no signs of joint inflammation or joint deformity, but limited the range of motion due to pain. Other physical examinations were unremarkable. Chest X-ray showed cardiomegaly (cardio-thoracic ratio 0.6), normal pulmonary blood flow, and no pulmonary congestion. He was admitted to the hospital for further investigations. Electrocardiogram showed right axis deviation and low QRS voltage. Echocardiogram showed moderate to severe TR with TRPG 80 mmHg, right atrium and right ventricle enlargement with D-shape LV, impaired RV systolic function, and no evidence of intra-cardiac shunt. PAH was diagnosed. Results of biochemical investigations are shown in Table 1. Non-invasive respiratory support and pulmonary vasodilator were given. He also received a single dose of 100 mg-thiamine intravenously and daily 300 mg of vitamin C *via* oral route. His symptoms improved dramatically the next day, he was able to wean himself off of respiratory support, and an echocardiogram revealed that his pulmonary hypertension had improved. He was discharged on the fifth day of admission. Nutrition counseling was given to the caregiver before discharge. Multivitamins, vitamin C, iron, and folate supplement were given and continued after discharge. Follow-up visit at 3 weeks after discharge showed improvement in the quality of dietary intake. The patient could reduce milk intake to four cartons per day but still had bottle feeding. He still refused fruits and vegetables, but took fresh fruit juice daily as a dietary source of vitamin C. No symptoms and signs of vitamin C deficiency were presented. Echocardiogram at 3 months after admission showed no pulmonary hypertension.

## Nutrient intake analysis

At diagnosis, dietary history was obtained from 24-h food recall by interviewing the caregiver. Nutrient intakes of both patients were analyzed from the dietary history by INMUCAL software, a computer program for the analysis of nutrients from the Thai food database, developed by the Institute of Nutrition, Mahidol University.^1^ The amount of nutrient intakes was compared with dietary reference intakes (DRIs) for Thais 2020 (2) as shown in Table 2. Inadequate intake was determined as the amount of nutrient intakes lower than 67% of the amount recommended in DRIs. Both patients had adequate protein intake but no vitamin C-containing food was taken. Many micronutrient intakes in Case 1 were inadequate. Case 2 showed inadequacy of vitamin C, iron, and copper intakes, but the amount of many micronutrients contained in UHT milk was over the DRIs.

**TABLE 2:** Nutrient analysis from dietary history at diagnosis compared with dietary reference intakes (DRIs) for Thai 2020.

Nutrient	DRIs for Thai	Case 1		Case 2	
		Intake	% DRIs	Intake	% DRIs
Protein (g/kg/day)	1.2	1.38	115	2.4	200
Vitamin A (mcg)	350	275.2	79	828.6	237
Vitamin B1 (mg)	0.6	0.246	41*	0.9	150
Vitamin B2 (mg)	0.6	0.59	98	4.59	765
Vitamin B6 (mg)	0.6	0.21	35*	3.0	500
Vitamin B12 (mcg)	1.2	1.74	145	7.05	586
Vitamin C (mg)	40	0	0*	0	0*
Calcium (mg)	800	131	16*	2222.1	278
Copper (mg)	1	0.243	24*	0.08	8*
Iron (mg)	6.6	4.03	61*	3.51	53*
Zinc (mg)	5.3^†^, 6.3^‡^	2.43	38*	8.29	276

^†^Age 3–5 years.

^‡^Age 6–8 years.

*Below 67% of DRIs.

## Discussion

This is a report of two patients with ASD who were diagnosed with PAH. Both patients had symptoms and signs of vitamin C with subclinical multiple micronutrient deficiencies including iron, zinc, and vitamin D. Growth parameters showed that they were not underweight, wasting, or stunting, which implied that they could obtain adequate calories for growth. Moreover, obesity was presented in case 2 as a result of excess calories from milk intake. However, the analysis of dietary history showed that both patients had inadequate micronutrient intakes (Table 2). ASD children are more likely to have feeding behavior problems such as picky eater or emotional eating even if they grow beyond the toddler period (3, 4). Feeding behavior problems lead to inappropriate dietary intake and risk of multiple micronutrient deficiencies. The repetitive dietary pattern was presented in both cases which leads to limit micronutrient intakes despite adequate protein and energy consumption. One of our cases had excess milk intake *via* bottle feeding. This feeding habit resulted in a decreased intake of other foods and limited diet variation. Our results showed the inadequacy of micronutrient intakes in both patients caused by limited intakes of fruits and vegetables as a dietary source of vitamin C, and meat as a dietary source of trace elements (iron, zinc, and copper). We also found a low 25-OHD level which implied that they had vitamin D deficiency. Vitamin D intake was not analyzed due to the limited database of vitamin D content in Thai foods. We speculated that limited outdoor activity may be the cause of vitamin D deficiency in both cases, because the presentation occurred during the COVID-19 pandemic and the lockdown measures in Thailand.

Typical presentations of vitamin C deficiency are fatigue, weakness, loss of appetite, perifollicular petechial hemorrhage, bruising and ecchymoses, bleeding per gum, and refusing to walk or limping due to bone pain (1). PAH is an unusual presentation of vitamin C deficiency. Table 3 shows the summary of case reports of vitamin C-associated PAH in children from previous literature. The diseases occurred among children with normal weight, underweight, and obesity. All cases were male subjects and most of them had developmental or behavioral problems.

**TABLE 3:** Previous case reports of pulmonary arterial hypertension associated with vitamin C deficiency.

Study	Patient characteristics	Growth status	Typical presentations of vitamin C deficiency	Clinical presentations of PAH	Vitamin C level	Combined micronutrient deficiency
Duvall et al. (6) USA	9-year-old boy with ASD	Wt 45 Kg (96^th^ percentile)	Limping	Dry cough, breathing difficulty	Undetectable	Vitamin B1, B6, B12 Vitamin D
Dean et al. (12) USA	6-year-old boy	NA	Lower extremities pain	Cardiac arrest	Undetectable	Vitamin A Vitamin B6
Ichiyanagi et al. (13) Japan	3-year-old boy with autism	Wt 12.3 Kg Ht 95 cm BMI 13.6 Kg/m^2^	Unstable gait	Heart failure	Undetectable	NA
Abe K, et al. Hawaii (5)	7-year-old boy with ASD	Wt 18.1 Kg (< 1^st^ percentile) Ht 130 cm (78^th^ percentile) BMI 12.7 Kg/m^2^	Polyarthalgia, gingival bleeding, perifollicular petechiae	Fatigue, tachycardia	Undetectable	Iron
Tan et al. Malaysia (14)	7-year-old boy with ASD	NA	Refuse to walk	Unexplained sinus tachycardia, desaturation	Undetectable	NA
Case presentation 1	6-year-old boy with ASD	Wt 20 Kg (WAZ –0.23) Ht 112 cm (HAZ –0.86) BMI 15.9 Kg/m^2^	Knee swelling, refuse to walk, corkscrew hair	Progressive dyspnea	Undetectable	Iron Vitamin D
Case presentation 2	5-year-old boy with ASD	Wt 30.2 Kg (WAZ 3.01) Ht 115 cm (HAZ 0.2) BMI 22.8 Kg/m^2^	Refuse to walk,	Progressive dyspnea, cardiomegaly	Undetectable	Iron Zinc Vitamin D Folate

PAH, pulmonary arterial hypertension; ASD, autistic spectrum disorder; Wt, weight; Ht, height; BMI, body mass index; NA, not available.

The pathogenesis of PAH caused by vitamin C deficiency was proposed in previous literatures (5, 6). First, vitamin C involves in nitric oxide (NO) synthesis which is responsible for pulmonary vascular dilatation. Low NO level in vitamin C-deficient subject causes an increase in pulmonary vascular resistance, which leads to PAH. Second, vitamin C is a cofactor of the prolyl hydroxylases family, which regulates a hypoxia-inducible factor (HIF). Low vitamin C levels cause an increase in HIF, which leads to an increase in the production of vasoconstriction factors (7). Lastly, some evidence suggest a link between changes in reactive oxygen species (ROS) and the development of PAH (8). Vitamin C has an antioxidant effect and involves in ROS scavenging. Therefore, vitamin C deficiency causes an imbalance in ROS, which results in PAH.

Although vitamin C involves in many mechanisms related to pulmonary arterial pressure, vitamin C deficiency alone may not a cause of pulmonary arterial hypertension. Most patients with vitamin C deficiency do not present with PAH. Instead, combinations of multiple micronutrient deficiencies, including vitamin D, iron, and flavonoids, are responsible for the pathogenesis of PAH (9). Our case presentations also exhibited vitamin D and iron deficiency. Previous studies found that the majority of case reports of vitamin C-associated PAH were from ASD children rather than typically developing children, and that they had multiple micronutrient deficiencies (Table 3). As ASD children have more severe eating problems and inappropriate dietary intakes, they may have more severe vitamin C deficiency and other micronutrient deficiencies which lead to the development of PAH.

In addition to vitamin C, children with ASD were reported to have inadequate micronutrient intakes, including vitamin D, calcium, iron, zinc, and fiber, compared to healthy children (10, 11). To prevent nutrient deficiencies among ASD children, nutrition counseling should be provided. The nutritional status and dietary intakes of children with ASD should be regularly monitored. Dietary intervention and micronutrient supplements should be appropriately provided. Medical formulas fortified with multiple micronutrients may be used as an optional source of micronutrients. In our cases, both patients could not modify their eating behaviors in a short follow-up time. Therefore, micronutrient supplementation was given until they could increase diet variety and appropriate food intake.

## Conclusion

Our case study shows ASD patients with vitamin C deficiency who had a clinical presentation of PAH. We proposed that severe vitamin C deficiency combined with other micronutrient deficiencies may contribute to the pathogenesis of PAH. Feeding behavior problems that are commonly found in ASD children can lead to an increased risk of multiple micronutrient deficiencies. Dietary counseling and regular nutrition assessment among children with ASD are needed to prevent nutrient deficiencies which may lead to life-threatening complications.

## Data availability statement

The raw data supporting the conclusions of this article will be made available by the authors, without undue reservation.

## Ethics statement

The studies involving human participants were reviewed and approved by Human Research Ethic Committee, Faculty of Medicine Ramathibodi Hospital, Mahidol University. Written informed consent to participate in this study was provided by the participants or their legal guardian/next of kin.

## Author contributions

WS, OD, and SS were pediatric nutritionists who were responsible for nutrition management in both patients in pediatric ward and follow-up visits. WS did the nutrient intake analysis by INMUCAL software and wrote the first draft of the manuscript. OD and SS revised the manuscript. All authors designed case report writing and approved the manuscript before submission.

## Footnotes

1
https://inmu2.mahidol.ac.th/inmucal/
